# Sister Mary Joseph Nodule as a First Manifestation of a Metastatic Ovarian Cancer

**DOI:** 10.1155/2016/1087513

**Published:** 2016-08-18

**Authors:** Giannina Calongos, Mai Ogino, Takatoshi Kinuta, Masateru Hori, Tatsuo Mori

**Affiliations:** Department of Obstetrics and Gynecology, Meiwa General Hospital, Nishinomiya 663-8186, Japan

## Abstract

A 76-year-old female presented to our hospital with a 2 cm firm, nontender, protuberant umbilical nodule. She received treatment with antibiotics for suspected granuloma, with no improvement after two months. High levels of CA125 as well as an ovarian cyst and intrathoracic and intra-abdominal lesions on imaging studies made us suspect an ovarian cancer with a Sister Mary Joseph nodule (SMJN) and other metastases. A bilateral salpingo-oophorectomy and umbilical and omentum tumor resections were performed and a metastatic ovarian serous adenocarcinoma was diagnosed by histopathology. After surgery, the patient received chemotherapy with paclitaxel, carboplatin, and bevacizumab; however paclitaxel allergy was observed. As a result, chemotherapy continued with carboplatin and bevacizumab every three weeks for a total of 6 courses. Currently, she is still undergoing treatment with bevacizumab and CA125 levels have been progressively decreasing. SMJN is a rare umbilical metastasis which needs to be considered as a differential diagnosis in the presence of an umbilical tumor for prompt treatment initiation.

## 1. Introduction

Sister Mary Joseph nodule (SMJN) is a rare umbilical lesion resulting from an intra-abdominal and/or pelvic malignancy. It was named after Sister Mary Joseph, a surgical assistant to Dr. William J. Mayo, who noted the association between the presence of an umbilical nodule and an intra-abdominal malignancy [1]. Its incidence is 1%–3% of all intra-abdominal or pelvic malignancies [2]. Gastrointestinal malignancies, most commonly gastric, colon, and pancreatic, account for about 52% of cases and gynecological cancers, most commonly ovarian and uterine, account for about 28% of the underlying sources [3]. Also, 15–29% of all cases have an unknown origin [4]. The mechanism of tumor spread to the umbilicus is poorly understand as it seems to be lymphatic, vascular, contiguous, or via embryologic remnants in the abdominal wall [5].

Here, we present a case of SMJN as an ovarian cancer metastasis, its diagnosis, treatment and follow-up.

## 2. Case Presentation

**Figure 1:**
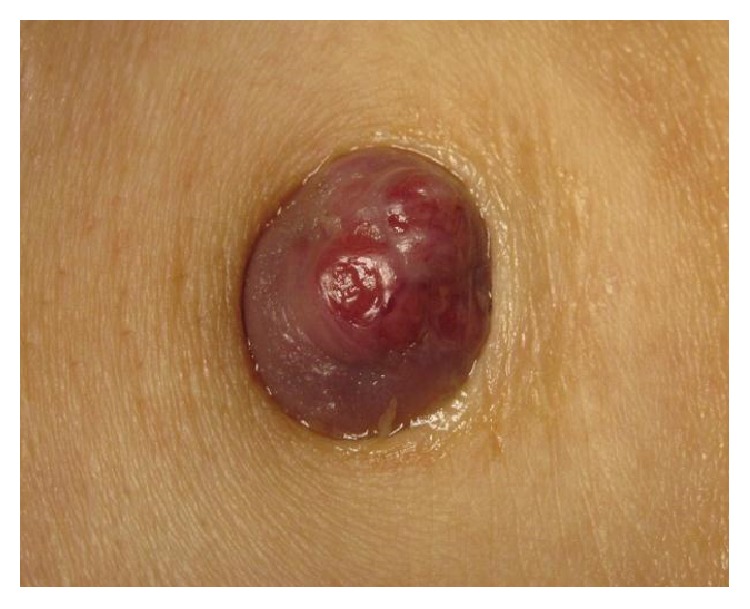
A 2 cm firm, protuberant, ulcerated tumor was observed in the umbilicus.

A 76-year-old female with a one-month history of a rapidly enlarging and friable umbilical tumor presented to our hospital for a surgery consult. She had a past history of hypertension, hyperlipidemia, and tubal ligation. Her family history was not relevant. Physical examination showed a 2 cm firm, nontender, protuberant umbilical nodule (Figure 1). She was diagnosed with telangiectatic granuloma and received treatment with topical antibiotics; however, after two months no improvement was noted.

**Figure 2:**
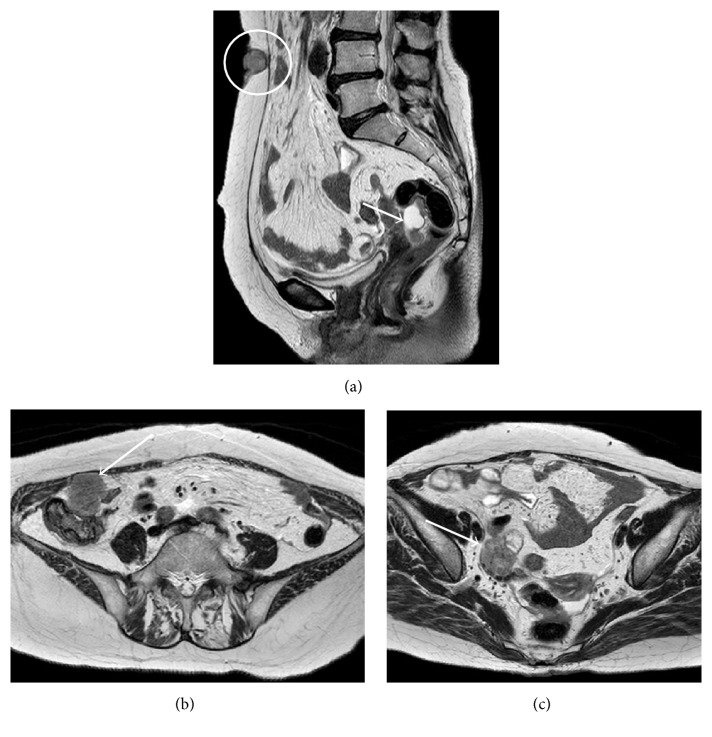
MRI images showed tumors in the ovary (a, →) and umbilicus (a, circle). Also intra-abdominal nodular lesions were noticed (b, c →).

**Figure 3:**
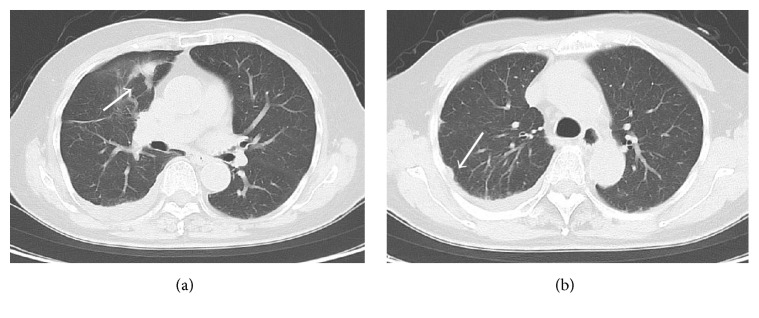
CT images showed nodular lesions in the right lung (a, b →).

With the suspicion of a malignant umbilical tumor, blood tests were performed and revealed high levels of CA125 (over 500 U/mL). Biopsy was proposed for a definitive diagnosis; however it was not pursued per patient's request. An upper and lower gastroenterological endoscopy was performed and showed no major abnormalities. Gynecology was consulted for further evaluation and a 2 cm ovarian cyst was noted on a transvaginal ultrasound. Imaging by CT and MRI confirmed the diagnosis and showed intra-abdominal and right lung metastatic lesions (Figures 2 and 3).

**Figure 4:**
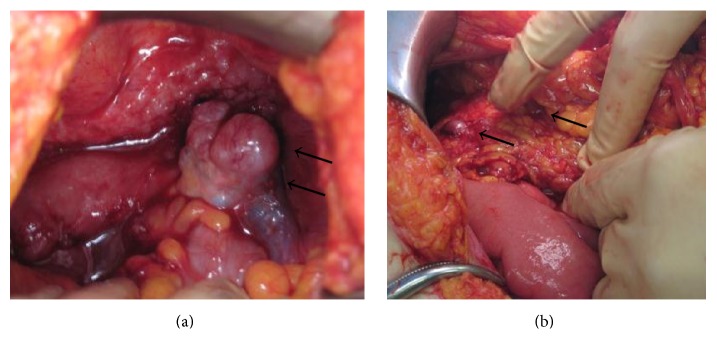
During surgery, a cystic lesion of 2-3 cm in the right ovary and ascites were noted (a →). Also, intra-abdominal and omentum metastatic lesions were observed (b →).

With the suspected diagnosis of a metastatic ovarian cancer, an exploratory laparotomy was performed. A cystic lesion of approximately 2-3 cm was seen on the right ovary and a small amount of ascites was noted (Figure 4(a)). Moreover, intra-abdominal and omentum metastatic lesions as large as 5 cm were observed (Figure 4(b)). Millet-seed sized lesions were noticed in the anterior and posterior uterine walls and the vesicouterine and Douglas pouches. As not all metastatic lesions were able to be resected, a bilateral salpingo-oophorectomy and umbilical and omentum tumor resections were performed. The histopathological diagnosis was consistent with an ovarian serous adenocarcinoma, which was also observed in the umbilical and omentum tumor. As a result, the conclusive diagnosis was a stage IV ovarian cancer (FIGO ovarian cancer staging).

The postoperative course was uneventful and the patient was discharged 10 days after surgery. Chemotherapy with paclitaxel, carboplatin, and bevacizumab was started one month after hospital discharge; however paclitaxel allergy (skin rash) was observed. As a result, chemotherapy continued with carboplatin and bevacizumab only, every three weeks for a total of 6 courses. Currently, the patient has monthly follow-ups with blood tests (tumor markers) and vaginal ultrasounds. CA-125 levels have been progressively declining, though they are still not under normal limits. The patient will continue with 10 additional chemotherapy courses with bevacizumab.

## 3. Discussion

Umbilical tumors are rare and can be classified as benign or malignant. Benign causes include umbilical hernia, granuloma, abscess, mycosis, and eczema. Malignant tumors can be either primary or metastatic [2, 6]. The presentation of a SMJN can be quite variable ranging from a hard and irregular nodule to a soft and painfully ulcerated mass [7]. On physical examination, its appearance is often misleading because the skin overlying the lesion can be normal or erythematous [8]. Previous reports showed that 60% of umbilical nodules were benign [9]. As a result an umbilical nodule may be present for several months before the diagnosis of a malignancy is finally established [8]. Since other symptoms were not observed, the first diagnosis in this case was a granuloma and it took two months of noneffective treatment to suspect a malignant umbilical tumor.

When an umbilical nodule is found it is necessary to make an accurate histological diagnosis between primary and metastatic lesions. Fine needle aspiration cytology and core biopsy are proposed as simple, fast, accurate, and inexpensive diagnostic tools [9, 10]. Also, ultrasonographically, a solid hypoechoic mass in the umbilicus with irregular margins and without any signs of inflammation involving the adjacent tissue might suggest the diagnosis of a SMJN [8]. Biopsy was recommended; however, the patient declined due to the risk of bleeding from the umbilical tumor.

Previous studies showed that, among umbilical malignancies, 88% originated outside the umbilicus and 12% were primary skin tumors. The mean age of diagnosis is approximately 50 years, with a range of 18–87 years. Moreover, women are more likely to have malignant tumors affecting the umbilicus [11]. Although SMJN is most commonly associated with gastrointestinal malignancies, in this case both upper and lower endoscopies did not provide major findings. Histologically, a metastatic umbilical tumor usually reveals an adenocarcinoma; however, sarcomas, mesotheliomas, and melanomas have also been reported [4]. In this case the final pathological diagnosis was an ovarian serous adenocarcinoma with intra-abdominal and thoracic metastases.

SMJN is considered a late manifestation of a malignant process and represents an advanced stage of the disease [8]. Mean life expectancy is 2–11 months without treatment [2]. Recent reports have proposed an aggressive treatment combining surgical excision, radiotherapy, and chemotherapy with a mean survival of 17.6–21 months. However, as the disease is usually advanced and metastatic, often only palliative treatment is offered [2, 4, 10]. The patient in this case received surgery treatment and is still undergoing chemotherapy without complications. CA125 levels have been decreasing which made us suspect a good response for the treatment; however, strict follow-up is necessary.

In conclusion, the presence of SMJN is a rare and often poor prognostic sign of a disseminated malignancy. SMJN needs to be considered as a differential diagnosis of an umbilical nodule in order to make a prompt identification of the primary lesion.
